# Apixaban for Primary Prevention of Venous Thromboembolism in Patients With Multiple Myeloma Receiving Immunomodulatory Therapy

**DOI:** 10.3389/fonc.2019.00045

**Published:** 2019-02-26

**Authors:** Robert Frank Cornell, Samuel Z. Goldhaber, Brian G. Engelhardt, Javid Moslehi, Madan Jagasia, Daryl Patton, Shelton Harrell, Robert Hall, Houston Wyatt, Greg Piazza

**Affiliations:** ^1^Division of Hematology and Oncology, Department of Medicine, Vanderbilt University Medical Center Nashville, TN, United States; ^2^Division of Cardiovascular Medicine, Brigham and Women's Hospital, Harvard Medical School Boston, MA, United States; ^3^Cardio-Oncology Program, Vanderbilt University Medical Center Nashville, TN, United States; ^4^Vanderbilt Institute for Clinical and Translational Research Nashville, TN, United States

**Keywords:** Apixaban (Eliquis®), myeloma, anticoagulation (AC), immunomodualtors, venous thomboembolism

## Abstract

Immunomodulatory drugs (IMiDs), including thalidomide, lenalidomide, and pomalidomide, have improved survival of patients with multiple myeloma (MM). However, these therapies are associated with an increased risk of venous thromboembolism (VTE). Apixaban has been approved for treatment of acute VTE and for risk reduction of recurrent VTE following initial therapy. In this phase IV single-arm study (NCT02958969), we aim to prospectively evaluate the safety and efficacy of apixaban for primary prevention of VTE in patients with MM. The primary efficacy objective of this trial is to determine the rate of symptomatic VTE, including deep vein thrombosis (DVT) and pulmonary embolism (PE), over 6 months. The primary safety objective is to determine the rate of major bleeding in MM patients receiving apixaban prophylaxis. If proven safe and effective, apixaban will emerge as a promising option for oral VTE prophylaxis in MM patients.

## Introduction

Multiple myeloma (MM) is a plasma cell malignancy characterized by clonal proliferation of malignant plasma cells in the bone marrow microenvironment, monoclonal protein in the blood or urine, and associated organ dysfunction (1). The 5-year survival rates of MM have nearly doubled during the past 3 decades (24.6% in 1975 vs. 52.4% in 2008) due to treatment advancements such as immunomodulatory/cereblon-binding drugs (IMiDs: thalidomide, lenalidomide, pomalidomide), proteasome inhibitors (PIs: bortezomib, carfilzomib, ixazomib), monoclonal antibodies targeting CD38 (daratumumab) and SLAMF7 (elotuzumab), among other emerging therapies (2).

### Multiple Myeloma and Thrombosis

MM has been associated with an increased risk of venous thromboembolism (VTE) (3, 4). Compared with the general population, two large population-based studies found the incidence of VTE to be higher in patients with MM (5, 6). In a study of over 4 million US veterans, VTE risk was 9-fold higher in patients with MM compared all other patients. In a Swedish study of over 18,000 patients with MM and matched controls, the risk of VTE was 7.5-, 4.6-, and 4.1-fold higher at 1, 5, and 10 years, respectively, in patients with MM.

The risk of VTE is further increased in the setting of IMiDs (Table 1) (3, 7). However, the mechanism by which IMiDs increase thromboembolic events is unknown (3, 7). A meta-analysis of >3,000 MM patients found the risk of VTE increased 2.6-fold with thalidomide and 8-fold when thalidomide was prescribed with dexamethasone (8). In newly diagnosed MM, patients treated with lenalidomide plus high-dose dexamethasone a high rate (26%) of VTE was observed (9). These early associations of increased VTE risk with IMiDs have led to the common practice of thromboprophylaxis in MM. Due to the increasing use of IMiD-based regimens, prevention of VTE has become a major management challenge during MM treatment.

**Table 1:** Rates of cardiovascular toxicities of proteasome inhibitors and immunomodulatory drugs.

**Therapy**	**Cardiac toxicity**	**Venous or Arterial thromboembolism**
**PROTEASOME INHIBITOR (PI)**		
Bortezomib (1)	0.4–7.6%	
Carfilzomib (2)	7–25%	
**IMMUNOMODULATORY DRUGS (IMiDs)**		
Thalidomide (3)		3–4%
Plus dexamethasone (4)		4–26%
Plus melphalan (5)		11–20%
Plus doxorubicin (6)		26–58%
Plus multi-agent		16–31%
Chemotherapy (7)		
Lenalidomide (8)		0–13%
Plus dexamethasone (9)		11–75%
Pomalidomide (10)		0–5%
**COMBINATION PI AND IMiD**		
Carfilzomib and Lenalidomide (11)	6–19%	10.3–13–3%
Carfilzomib and Thalidomide (12)	5–19%	7–30%
Ixazomib, Lenalidomide (13)	1–16%	2–8%

### VTE Prophylaxis Strategies in MM

VTE risk assessment in MM includes individual risk factors, such as obesity, previous VTE, immobilization, disease status, and therapeutic regimen (3). Several VTE prophylactic strategies have been evaluated in MM (10, 11). However, prospective clinical trials are rare, and the optimal thromboprophylactic regimen remains uncertain. A phase III trial of 667 patients with newly diagnosed MM (NDMM) treated with thalidomide-based regimens indicated that aspirin (100 mg daily) or low-dose warfarin (1.25 mg daily) showed efficacies similar to LMWH (enoxaparin, 40 mg daily) in reducing VTE. However, patients with high risk of VTE were excluded from this study (12). Another prospective study included 342 patients randomized to either low dose aspirin (100 mg daily) or LWMH (enoxaparin 40 mg daily) with NDMM who were undergoing treatment with lenalidomide and low-dose dexamethasone. Aspirin was as effective as LMWH in this study; however, patients in this trial were relatively young (<65 years), lacked risk factors for cardiovascular disease or thrombosis, and had no history of VTE (13). Based on these limited studies and the consensus opinion of experts in the field, the International Myeloma Working Group has made recommendations regarding VTE risk assessment and prevention of IMiD-associated thrombosis in patients with MM. While aspirin (81–325 mg/day) is recommended for low risk patients, low molecular weight heparin (LMWH) is suggested for patients determined to be at high-risk for VTE (3).

LMWH, such as dalteparin, enoxaparin, and tinzaparin, have been well-studied for secondary prevention of cancer-related VTEs. However, the use of LMWH is complicated by injection site discomfort and consequent poor adherence to the prescribed regimen (14). In addition, LMWH is contraindicated in patients with a history of heparin-induced thrombocytopenia (HIT) and introduces a small risk of inducing HIT in patients (15).

A growing body of literature supports the use of direct oral anticoagulants (DOACs), including apixaban, for VTE prophylaxis (16). Apixaban directly blocks Factor Xa, which inhibits coagulation by interfering with conversion of prothrombin to thrombin, and is largely metabolized in the liver (17). Compared with injectable thromboprophylactic regimens, such as enoxaparin, apixaban offers the advantages of oral administration and does not require laboratory monitoring (18).

Apixaban prophylactic strategies have been evaluated for prevention of post-operative VTE after hip and knee replacement (Table 2). Studies of arthroplasty patients suggest that 2.5 mg twice-daily apixaban is superior to enoxaparin for VTE prevention with similar bleeding event rates. Apixaban is currently FDA-approved for treatment of acute VTE and prophylactic use following hip and knee replacement surgery and for reducing the risk of stroke in patients with non-valvular atrial fibrillation (23, 24).

**Table 2:** Research supporting prophylactic use of apixaban after orthopedic surgery.

**Study**	**Patient #**	**Drugs tested**	**Study duration**	**VTE rate**	**Bleeding rate**
Lassen et al. (19)	1,238	Apixaban (2.5 mg bid) vs. enoxaparin (30 mg bid) or open-label warfarin (INR 1.8–3.0)	10–14 day treatment	9.0% vs. 15.6% vs. 26.6% *p* = 0.13	3.9% vs. 5.4% vs. 5.3% *p* = 0.02
Lassen et al. (20)	1,599 vs. 1,596	2.5 mg twice daily apixaban vs. 30 mg enoxaparin	10–14 day treatment, 6 month follow-up	9.0% vs. 8.8% *p* = 0.06	2.9% vs. 4.3% *p* = 0.03
Lassen et al. (21)	1,528 vs. 1,529	2.5 mg twice daily apixaban vs. 40 mg enoxaparin	10–14 day treatment	15% vs. 24% *P* < 0.001	4.0% vs. 5.0% *p* = 0.09
Lassen et al. (22)	1,949 vs. 1,917	2.5 mg twice daily apixaban vs. 40 mg enoxaparin	35 days	1.4% vs. 3.9% *P* < 0.001	4.8% vs. 5.0%

In the randomized trial, Apixaban after Initial Management of Pulmonary Embolism and Deep Vein Thrombosis (AMPLIFY-EXT), apixaban (2.5 or 5 mg twice daily) was evaluated vs. placebo after patients completed 6–12 months of anticoagulation (25). The rate of recurrent VTE was reduced from 8.8 to 1.7% in patients receiving placebo or apixaban, respectively. No differences were found between the two dose levels of apixaban for recurrence of VTE (1.7 vs. 1.7%). There were no significant differences in the risk of major hemorrhage between dose levels (0.2 vs. 0.1%) and this risk was similar to that seen in the placebo group (0.5%). Apixaban was also effective in preventing thromboembolism in patients receiving chemotherapy for advanced metastatic cancer (26). In this phase II trial, patients were randomized to receive 5, 10, or 20 mg once daily or placebo over 12 weeks. The rate of major and non-major bleeding was 3.1, 3.1, and 3.4% in the 5, 10 mg, and placebo groups, respectively. Risk of VTE was higher in the placebo group compared to all groups treated with apixaban (10.3 vs. 0%). Ten patients with metastatic MM were included in the apixaban cohorts (26).

Here, we describe our phase IV clinical trial (NCT02958969) that will further investigate the safety and efficacy of apixaban for VTE prophylaxis for patients with MM.

## Main Study Objectives

Our primary efficacy outcome is to assess the rate of symptomatic VTE over 6 months in patients with MM receiving IMiDs who are prescribed apixaban 2.5 mg orally twice daily for primary prevention of VTE. Accounting for patients receiving IMiDs at all phases of treatment, including maintenance, we hypothesize that the 6-month rate of symptomatic VTE will be <5% (27–30).

Our primary safety objective is to quantitatively assess the 6-month rate of major and clinically-relevant, non-major bleeding. We hypothesize that the 6-month rate of major and clinically-relevant, non-major bleeding in these patients will be ≤3% (31). We will also quantify the 6-month rate of myocardial infarction and stroke, which are known to occur in patients with MM receiving IMiDs (32).

## Methods

### Study Design

**Figure 1:**
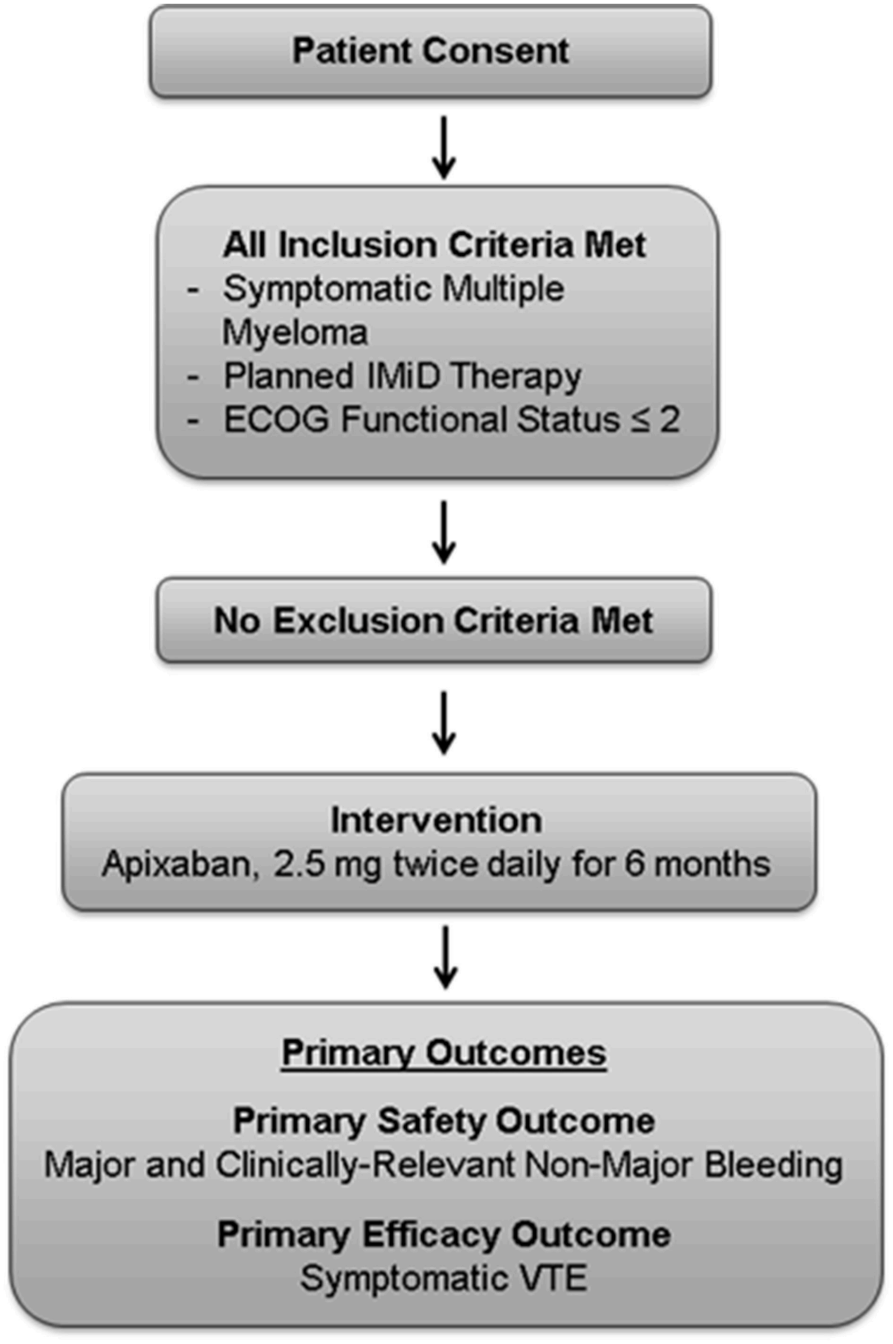
Study management scheme.

This is a phase 4, investigator-initiated U.S.-based, single-center, single-arm, open-label, proof-of-concept study (NCT02958969; Figure 1). Fifty patients will receive apixaban 2.5 mg orally twice daily for primary prevention of VTE for a planned 6 months. We project that this study will be completed in 24 months. The study population will include both male and female patients who are 18 years of age or older with MM defined according to the International Myeloma Working Group (IMWG) guidelines, and receiving IMiD-based therapy (33). Patients must have an Eastern Cooperative Oncology Group (ECOG) functional status ≤2. Patients will be receiving or starting IMiD therapy, with planned IMiD therapy for a minimum of 6 months. Patients will begin prophylactic anticoagulation within 3 weeks of enrollment. Patients will be instructed to stop aspirin prophylaxis while receiving apixaban. Inclusion and exclusion criteria are detailed in Tables 3, 4.

**Table 3:** Inclusion criteria.

Men and women Age > 18 years Current or prior diagnosis of symptomatic MM based on International Myeloma Working Group (IMWG) guidelines Starting or already receiving IMiD therapy with thalidomide [Thalomid], lenalinomide [Revlimid], or pomalidomide [Pomalyst] IMiD therapy given in the setting of newly diagnosed MM, relapsed MM, progressive MM, maintenance therapy/consolidation therapy as per IMWG criteria Patients must have had measurable disease as defined by at least one of the following: Serum M-protein ≥ 0.5 g/dL by serum electrophoresis (SPEP) Quantitative IgA (>750 mg/dl) Urinary M-protein excretion ≥ 200 mg/24 h Serum Free Light Chain (FLC) ≥ 10 mg/dL, with an abnormal light chain ratio Willing to provide written informed consent Eastern Cooperative Oncology Group (ECOG) functional status ≤ 2 Plan IMiD therapy for a minimum of 6 cycles

**Table 4:** Exclusion criteria.

Pregnant or breastfeeding Women of child-bearing potential unwilling or unable to use an acceptable method of birth control Any prior venous thromboembolism Contraindication to anticoagulant therapy Conditions for which serious bleeding may occur: Current or within 6 months: intracranial bleeding, intraocular bleeding, gastrointestinal bleeding, endoscopically-documented ulcer disease Current or within last month: head trauma or other major trauma, major surgery Current or within last 2 weeks: stroke, neurosurgical procedure Current: gross hematuria, major unhealed wound, major surgery planned during the trial period, intracranial mass, vascular malformation, or aneurysm, overt bleeding, blood dyscrasia CNS involvement of MM or other history of CNS malignancy Active and clinically significant liver disease Uncontrolled hypertension: systolic blood pressure >180 mm Hg or diastolic blood pressure >100 mm Hg Current endocarditis Requirement for ongoing anticoagulant therapy, including mechanical heart valve replacement and atrial fibrillation Severe valvular heart disease, including rheumatic heart disease and mitral stenosis Requirement for dual antiplatelet therapy or single agent antiplatelet therapy with clopidogrel, prasugrel, or ticagrelor Bioprosthetic heart valve replacement Requirement for aspirin >165 mg daily Hemoglobin < 9 mg/dL at time of screening Platelet count < 100,000/mm^3^ at time of screening Serum calculated creatinine clearance (CrCl) < 25 ml/m at time of screening Alanine aminotransferase or aspartate aminotransferase level > 2 times the upper limit of the normal at time of screening Total bilirubin level > 1.5 times the upper limit of the normal at time of screening Life expectancy < 12 months or hospice care Prisoners or subjects who are involuntarily incarcerated Subjects who are compulsorily detained for treatment of either a psychiatric or physical (e.g., infectious disease) illness Receiving concurrent non-FDA-approved or investigational agents or has received an investigational agent within the past 30 days prior to the first dose of study treatment Any condition, which in the opinion of the investigator, would put the subject at an unacceptable risk from participating in the study Any other medical, social, logistical, or psychological reason, which in the opinion of the investigator, would preclude compliance with, or successful completion of, the study protocol

### Primary and Secondary Study Outcomes

#### Efficacy Outcomes

As our primary efficacy outcome, we will assess the rate of symptomatic VTE, including deep vein thrombosis (DVT) and pulmonary embolism (PE), over 6 months. DVT will be diagnosed by ultrasonography as a non-compressible venous segment or segments or as a filling defect detected by computed tomographic (CT) venography, magnetic resonance (MR) venography, or contrast venography of the lower extremities from the popliteal vein or higher and all upper extremity DVTs will be captured including catheter-related. PE, subsegmental or greater, will be diagnosed by the presence of mismatched perfusion defects during ventilation perfusion scan, the presence of a pulmonary artery filling defect detected by contrast-enhanced chest CT, an intraluminal filling defect detected by invasive pulmonary angiography, or confirmation of PE at autopsy (25). Accordingly, as a secondary efficacy outcome, we will investigate the rate of MI and stroke at 6 months. Acute MI will be defined according to current standards (34). All strokes during the study will be assessed by imaging or autopsy and classified as primary hemorrhagic, non-hemorrhagic, infarction with hemorrhagic conversion, or unknown, as defined by the AHA/ASA (35).

#### Safety Outcomes

As our primary safety outcome, we will assess the rate of major and clinically relevant non-major bleeding over 6 months (Table 5). According to the International Society on Thrombosis (ISTH) classification, major bleeding is defined as overt bleeding that is associated with a decrease in hemoglobin of 2 g/dL or more, requiring the transfusion of 2 or more units of blood, occurring in a critical site, or contributing to death (36). ISTH classification defines clinically relevant non-major bleeding as overt bleeding that does not meeting the criteria for major bleeding, but is associated with medical intervention, surgical intervention, or interruption of the study drug. Secondarily, we will record all mortality at 6 months. Cause of death will be classified as related to cancer, myocardial infarction, pulmonary embolism, other cardiovascular causes, or due to other disease states.

**Table 5:** Definition of major and non-major bleeding events^a^.

**Major bleeding event**	**Clinically-relevant non-major bleeding event**
Acute clinically overt bleeding accompanied by one or more of the following: Decrease in hemoglobin of 2 g/dl or more Transfusion of 2 or more units of packed red blood cells Bleeding that occurs in at least one of the following critical sites: intracranial, intra-spinal, intraocular (within the corpus), pericardial, intra-articular, intramuscular with compartment syndrome, retroperitoneal	Acute clinically overt bleeding that consists of one or more of the following: Bleeding that compromises hemodynamics Bleeding that leads to hospitalization Subcutaneous hematoma larger than 25 cm^2^, or 100 cm^2^ if due to a traumatic cause Intramuscular hematoma documented by ultrasonography Epistaxis that lasted for more than 5 min, was repetitive (two or more within 24 h), or led to an intervention Spontaneous gingival bleeding that lasts for more than 5 min Hematuria that was macroscopic and spontaneous or lasted for more than 24 h after instrumentation of the urogenital tract Macroscopic gastrointestinal hemorrhage, including at least one episode of rectal blood loss, if more than a few spots on toilet paper Hemoptysis, if more than a few speckles in the sputum and not occurring within the context of PE Any other bleeding considered to have clinical consequences

a *Defined by International Society on Thrombosis and Haemostasis guidelines*.

### Study Monitoring

This study has been approved by the Institutional Review Board and is registered through ClinicalTrials.gov (NCT02958969). This study was designed in accordance with good clinical practice guidelines. The Vanderbilt University Medical Center Data Monitoring Committee is responsible for monitoring the progress and safety throughout the study. Adherence to the protocol will be assessed by pill count and calculated as the number of pills taken divided by the number of pills prescribed. Furthermore, subjects will be given a study diary to fill out at home each day. Patients will be asked to write down the time study drug is taken and any side effects. Study diaries will be collected at a regularly scheduled office visit at conclusion of the study period.

### Statistical Methods and Sample Size Estimation

A formal hypothesis test will not be conducted in this proof-of-concept single arm study. Sample size estimation will be based on the precision of the measurements using the 95% confidence interval (CI) method. A sample size of 50 patients generates a half-width of CI <10%. Based on the estimated 5% VTE incidence rate, the half-width CI will be <6%. Therefore, the precision will be effectively measured with the estimated CI.

### Statistical Analysis Plan

For this proof-of-concept study, we will calculate the 6-month rates of symptomatic VTE and major and clinically relevant non-major bleeding. We will also calculate 6-month rates of myocardial infarction and stroke. The primary analysis will be intention-to-treat.

Means, medians, and frequency distributions will be calculated for continuous variables. Number and percentages will be reported for binary and categorical variables. Differences between subgroups of interest will be examined using the chi-square or Fisher's exact test for binary and categorical variables and *t*-test or Wilcoxon Rank Sum for continuous variables, depending on the number of patients in each group. Subgroup analysis will include a comparison of newly diagnosed patients vs. those with recurrent or relapsed disease. Addition studies will compare patients based on the use of other concomitant medications (e.g., dexamethasone). All tests will be two-tailed and a *p* < 0.05 assumed to represent statistical significance. All analyses will be performed using SAS software.

## Conclusion and Discussion

Immunomodulator/cereblon-binding agents comprise the therapeutic foundation of therapy for MM at all phases of therapy. While these agents are generally well tolerated, their increased risk of VTE presents a clinical challenge. Further compounding this challenge is the lack of rigorous, prospective trials of thromboprophylactic regimens for VTE prevention in these vulnerable patients (16, 37). The prophylactic efficacy of apixaban in cancer is under evaluation in several clinical trials (i.e., NCT02048865, NCT02366871). This study will specifically assess the efficacy and safety of apixaban for primary prevention in MM. If successful, this regimen has the potential to greatly improve VTE prophylaxis options for patients with MM.

## Ethics Statement

This study was carried out in accordance with the recommendations of the Vanderbilt University Medical Center Institutional Review Board with written informed consent from all subjects. All subjects gave written informed consent in accordance with the Declaration of Helsinki. The protocol was approved by the Vanderbilt University Medical Center Institutional Review Board and Scientific Review Committee.

## Author Contributions

RC, SG, JM, and GP developed the Research Concept, designed, and wrote the protocol. RC, SG, JM, GP, MJ, DP, SH, RH, BE, and HW critically reviewed the manuscript at all steps of developed. RC, JM, MJ, DP, SH, RH, BE, and HW enrolled and treated patients and completed all study required training and protocol requirements.

### Conflict of Interest Statement

The authors declare that the research was conducted in the absence of any commercial or financial relationships that could be construed as a potential conflict of interest.
